# Traumatic terson syndrome with a peculiar mass lesion and tractional retinal detachment: a case report

**DOI:** 10.1186/s12886-024-03407-8

**Published:** 2024-04-08

**Authors:** Yung-Chen Lin, Chung-Ting Wang, Kuan-Jen Chen, Hung-Da Chou

**Affiliations:** 1 College of Medicine, Chang Gung University, Taoyuan, Taiwan; 2 Department of Ophthalmology, Linkou Branch, Chang Gung Memorial Hospital, 5 Fuxing Street, Guishan District, 333423 Taoyuan, Taiwan

**Keywords:** Terson syndrome, Retinal detachment, Epiretinal membrane, Vitreous hemorrhage, Proliferative vitreoretinopathy

## Abstract

**Background:**

To report a case with bilateral Terson syndrome presented with a unique mushroom-like mass lesion on the optic disc along with proliferative vitreoretinopathy and tractional retinal detachment.

**Case presentation:**

A 33-year-old man was injured during a traffic accident and had diffuse brain swelling and intraocular hemorrhage. Poor vision in both eyes was noted after the patient regained consciousness. B-scan ultrasonography showed extensive vitreous opacity with a posterior vitreous detachment and without obvious retinal detachment. Vitrectomy was performed in both eyes five months after the accident. After clearing up the vitreous opacity, a peculiar pigmented mushroom-like mass lesion was noted in the posterior pole and had severe adhesion to the underneath optic disc. Extensive multilayered peripapillary epiretinal membrane was found covering the posterior pole and led to tractional retinal detachment around the macula. The mass was presumed to be an organized vitreous hemorrhage originated from the optic disc. The extensive and adherent epiretinal membrane together with the mass lesion were removed as much as possible and silicon oil was injected for tamponade. However, in the right eye, the retina redetached under silicon oil, whereas in the left eye, his vision improved to 20/100.

**Conclusions:**

Terson syndrome usually has a favorable prognosis but may be complicated by proliferative vitreoretinopathy and tractional retinal detachment. Careful monitoring is warranted and early vitrectomy should be considered in cases suspecting additional pathologies.

**Supplementary Information:**

The online version contains supplementary material available at 10.1186/s12886-024-03407-8.

## Background

Terson syndrome was first described by Moritz Litten in 1881 as vitreous hemorrhage caused by subarachnoid hemorrhage. It was named by Albert Terson in 1900 and the initial definition was acute subarachnoid hemorrhage accompanying vitreous hemorrhage [1]. In recent years, the definition has been broadened to intraocular hemorrhages in combination with any type of acute intracranial hemorrhage [2]. Most of the Terson syndrome cases are caused by intracranial aneurysm rupture, while only 3% are related to trauma [3]. 

The pathogenesis of Terson syndrome is still contentious and the origin of intraocular hemorrhage was extensively debated. One possible explanation of Terson syndrome was that the ruptured intracranial aneurysm or a traumatic force to the head hastily increases the intracranial pressure, then the pressure was conducted by the optic nerve sheath to the intraocular space and caused hemorrhage [4]. 

Despite the acute initial vision loss, the prognosis is generally favorable in Terson syndrome. More than 80% of individuals could achieve a final visual acuity of 20/50 or better without surgical intervention [2]. Therefore, if no vision-threatening intraocular pathology is noted, a “wait and see” strategy is generally reasonable.

Nevertheless, in some circumstances, Terson syndrome may be complicated with serious conditions including retinal detachment, proliferative vitreoretinopathy, epiretinal membrane formation, retinal folds, and even macular holes. These pathologies possibly are the results of glial and retinal pigment epithelial cell proliferation, which may lead to fibrotic tissue formation and retinal deformation [4]. Here we report a tricky case of traumatic Terson syndrome with a unique presentation of the papillary mass lesion and drawstring bag-like tractional retinal detachment.

## Case presentation

A 33-year-old healthy gentleman was injured while riding a scooter without wearing a helmet. He had poor consciousness (E1V1M5) upon triage and brain computed tomography showed diffuse brain swelling, subarachnoid hemorrhage, and subdural hemorrhage. One month after the trauma, he regained consciousness and started to notice poor vision in both eyes. Upon ophthalmic examination, the vision was only with light perception in both eyes, the intraocular pressure was within the normal range, and the light reflexes were normal with no relative afferent pupillary defect. The lenses were clear but severe vitreous opacity obscured the viewing of both fundi. B-scan ultrasonography showed diffuse vitreous opacity with posterior vitreous detachments and no retinal detachment in both eyes, therefore close observation was suggested.

**Fig. 1:**
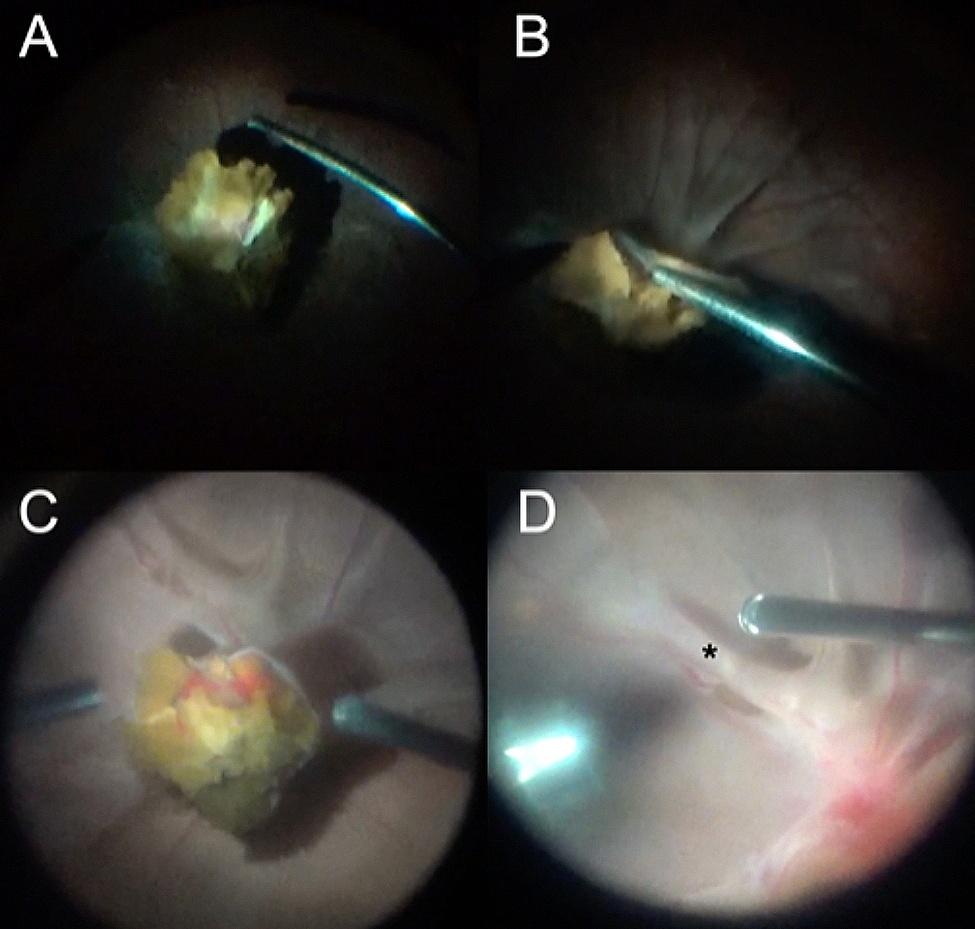
Intraoperative images of peculiar optic disc mass lesions and tractional retinal detachment in bilateral Terson syndrome. **(A, B)** A yellowish-pigmented lesion can be seen after core vitrectomy in the right eye. Severe retinal folds can be appreciated in **(B)**. **(C)** During vitrectomy for the fellow eye, a similar lesion was found. **(D)** After removing the mass lesion, retinal folds were noted around the macula (asterisk)

Three months later, the vitreous opacity remained, and the follow-up ultrasonography showed a stationary condition. After discussing with the patient and family, a vitrectomy was scheduled but later postponed due to an episode of hospital-acquired pneumonia. During the treatment of pneumonia, the patient received systemic corticosteroids (fludrocortisone acetate, administered orally 0.2 mg per day for eight days to address salt-wasting syndrome). Five months after the trauma, a vitrectomy in the right eye was arranged (Video 1). During the operation, a grade IV vitreous opacity was noted, and a careful vitrectomy was conducted. After clearing up most of the vitreous opacity, a peculiar brownish mass lesion was found covering the posterior pole, and the optic disc and macula could not be identified (Fig. 1A and B). Presumed to be an organized vitreous hemorrhage, the brownish mass was carefully removed by microforceps. During the process, severe adhesion of the mass lesion with the underlying retina was noted. After the successful removal of the mass, the optic disc could be seen, which was located directly under the mass.

Then, it was found that there was an extensive epiretinal membrane around the optic disc. These epiretinal membranes were thick, glutinous, multilayered, and very adherent to the underlying retinal tissue, therefore extremely difficult to remove (Video 1). An additional file explains Video 1 and Video 2 [see Additional file 1]. The perimacular traction related to the dense epiretinal membrane had caused a relatively flat tractional retinal detachment 360 degrees around the macula and formed a pouch-like configuration, which obscured direct viewing of the macula. After maximal efforts in peeling the epiretinal membrane, the macula could finally be visualized.

However, severe retinal folding around the macula persisted and could not be flattened even with the assistance of heavy liquids; therefore, laser coagulation was applied around the macula, and 5000 centistoke silicon oil was injected for long-term tamponade. Intraocular bleeding was controlled by diathermy.

Two days later, surgery was performed on the fellow eye (Video 2). Very similar findings were noted, with a dense vitreous opacity, peculiar brownish mass-lesion on the optic disc, and multilayered epiretinal membrane around the optic disc and covering the macula (Fig. 1). Fortunately, the macula could still be seen and there was no pouch-like tractional retinal detachment around the macula. Epiretinal membrane peeling was done to release the traction and 5000 centistoke silicon oil was used for tamponade after meticulous cauterization.

**Fig. 2:**
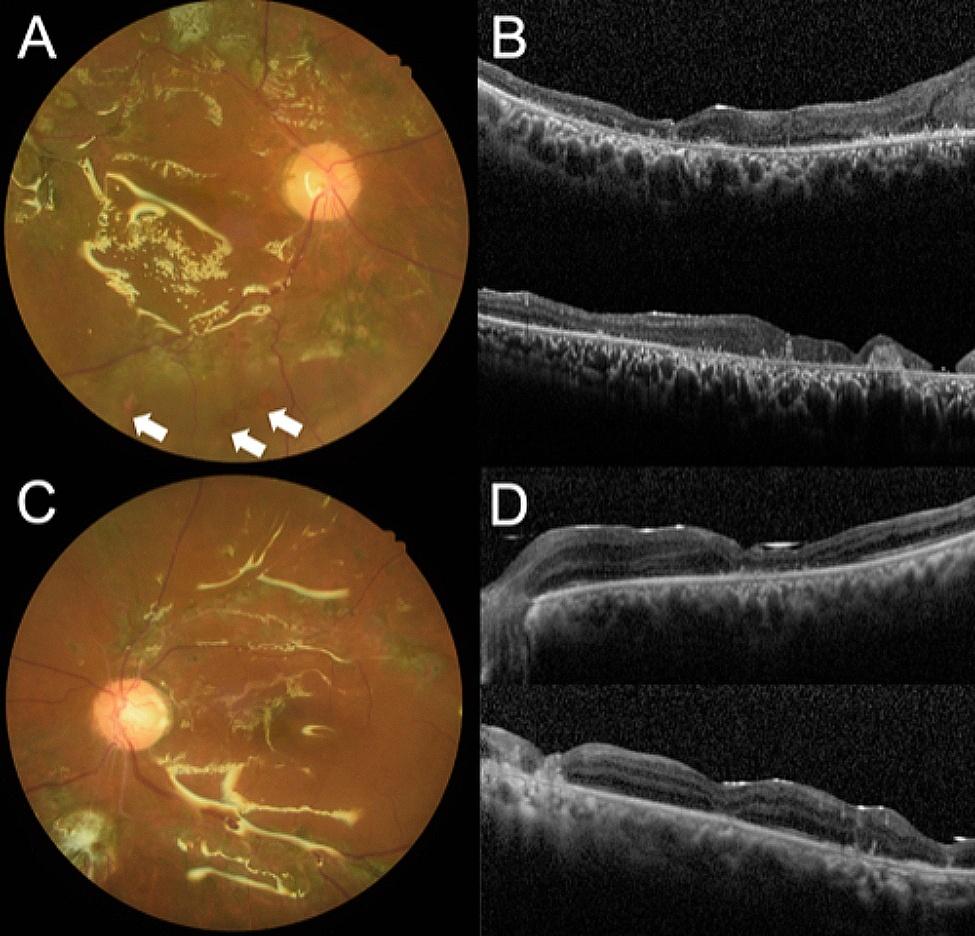
Postoperative color fundus photographs and optical coherence tomography images. **(A)** Inferior retinal breaks (arrows) and redetachment were noted in the right eye despite silicon oil tamponade. **(B)** The macula was atrophic. **(C)** The retina was well-attached in the left eye after silicon oil removal and the macula was relatively preserved **(D)**

Eight months after the surgery, inferior redetachment was noted in the right eye despite silicon oil tamponade and vision remained poor at hand motion (Fig. 2A and B). In the left eye, the retina was well-attached after silicon oil and cataract extraction six months after the initial surgery (Fig. 2C and D). His vision in the left eye improved to 20/100.

## Discussion and conclusions

The pathophysiology of Terson syndrome is still not clearly understood. Currently, there are two main theories: the glymphatic reflux theory suggests that the glymphatic system is the only extravascular anatomical channel that can communicate the subarachnoid space and the retina, which could be the blood-spreading route. When intracranial pressure is higher than intraocular pressure, subarachnoid blood may enter the globe via the glymphatic system and could not reflux back [5]. However, the glymphatic system within the subarachnoid space and retinal vessels has not been confirmed [6]. 

The retinal venous congestion theory suggests that the dilation of the optic nerve sheath, which is due to acute raised intracranial pressure and effusion of the cerebrospinal fluid, will compress the central retinal vein, block the drainage of venous blood from the retina, and trigger venous hypertension and stasis in the end. Blockade of the veins shuts down retinal microcirculation and leads to the intraocular venous stasis and hemorrhage observed in Terson Syndrome [6, 7]. Nevertheless, central retinal vein occlusions usually present as flame-shaped hemorrhage, while in Terson syndrome dome-shaped hemorrhage are usually observed.

The case we report had a mass-like lesion above the optic disc. Puri et al. reported a case of organized vitreous hemorrhage masquerading as a melanocytoma of the optic disc [8]. The mass-lesion presented in a patient with retinal detachment who underwent scleral buckling followed by vitrectomy and subsequent lensectomy due to vitreous hemorrhage and lens staining. The biopsy showed that the optic disc mass-lesion was a mixture of hemosiderin-laden macrophage accumulation and proliferation of melanocytes, which caused the lesion seemingly pigmented. Furthermore, the lesion had a “capsule” composed of retinal pigment epithelial cells and fibroblast-like cells with a sparse collagenous matrix, and there was no evidence of neoplasia [8]. To the best of our knowledge, no other similar published cases exhibit such peculiar mass-like lesions. Based on the similarity of the clinical presentation, we believe that the mass lesions noted in our case share the same nature as the reported case by Puri et al.

Proliferative vitreoretinopathy (PVR) likely played a key role in the unusual tractional retinal detachment in the macular region. The acute venous engorgement and stasis, combined with the acute plethora of ophthalmic arteries, may induce the retinal capillaries to rupture and cause longstanding intraretinal hemorrhages and general intraocular asphyxia. The resulting ischemic PVR can form pre-retinal neovascularization and fibrovascular membranes and further result in tractional retinal detachment as we had observed in our case [9, 10].

Following the pneumonia episode, the patient received systemic oral corticosteroids to treat salt-wasting syndrome. It was thought that corticosteroids might reduce the amount of intraocular inflammation and the related PVR. However, a recent meta-analysis of randomized controlled trials showed that following rhegmatogenous retinal detachment surgery, adjuvant systemic or intraocular corticosteroids only prevented the recurrence of PVR in PVR grades A and B but not in grade C patients [11]. Although the present case was not rhegmatogenous retinal detachment in nature, he demonstrated PVR grade C changes including thick epiretinal membrane, star-folds, and tractional retinal detachment. The course of corticosteroids might have partially decreased or postponed the PVR but was unlikely sufficient to prevent the subsequent complication entirely.

Terson syndrome resulting from subarachnoid hemorrhage often resolves spontaneously and has a good prognosis. However, in complicated cases, Terson syndrome may combine with other concomitant pathologies. A watchful follow-up and prudent serial ocular ultrasound are needed with particular attention to the formation of epiretinal membrane or retinal detachment. Lastly, in the current era of small-gauge vitrectomy, earlier intervention should be considered if no spontaneous recovery is observed, or complex pathologies are noted.

### Electronic supplementary material

Below is the link to the electronic supplementary material.

Supplementary file 1. Video 1. The surgery was performed on the right eye. We exerted maximum effort to delicately remove the vitreous opacity, the mass lesion, and the epiretinal membrane.

Supplementary file 2. Video 2. Two days later, we performed surgery on the left eye. We encountered similar findings to those in the right eye. We successfully removed the vitreous opacity, the mass lesion, and the epiretinal membrane.

## Data Availability

The data is available from the authors upon reasonable request. Please contact to Hung-Da Chou for access to the detailed raw data.
